# Argumentation, leadership, and curriculum

**Published:** 2017-06-30

**Authors:** Marcel D’Eon

**Affiliations:** 1 University of Saskatchewan, Saskatchewan, Canada

In volume 8(1) Litalien^1^ wrote about the value of thinking deeply about and seriously debating some of the more significant issues in health care. In this issue, Leontowicz bluntly assesses the ability of medical students to argue productively as largely wanting. Thankfully, Dr. Epstein has contributed an illuminating commentary to accompany the Leontowicz and Litalien letters boldly stating: “[…] the topic of argumentation as broadly conceived has a potential place in each of the seven CanMEDS roles.”

Two timely articles crossed my desk both of which are relevant to the leader role in general and the topic of argumentation in particular. Writing about the medical education enterprise, Casiro and Regehr^2^ assert, “effective academic governance is critical to effective curriculum delivery.” In exploring the health care system, Gonzalo et al.^3^ make the claim: “health systems are beginning to demand that providers have ‘systems-ready’ knowledge, attitudes, and skills” which means that “medical schools … modify their goals and prepare physicians to practice flexibly within teams and effectively contribute to the improvement of health care delivery.” Implied in these two recent calls to action is effective argumentation, a process that cooperatively seeks not to win but to find the best way forward. Unfortunately, very little curricular time if any is devoted to teaching and learning the dispositions and skills needed to argue effectively.

In the few years that I have included argumentation as a part of courses I taught, I have learned what Litalien and Leontowicz have told us. They seem to be saying that students are generally reluctant to engage in debate about fundamental pillars of medical practice (I think because they are preoccupied with memorizing what they have been told is important material for a battery of exams), and when they participate in argumentation it seems that are not well prepared and argue poorly. That is why the Structured Controversy^4^ activity is so valuable in teaching both the inner dispositions and the skills needed for cooperative argumentation. Epstein addresses these needs more thoroughly in his commentary. Perhaps the articles in this issue will spark some productive debate and substantive policy considerations.

Heading up this issue of the CMEJ is an interesting study titled “Relationship between Canadian medical student career interest in emergency medicine and post-graduate training disposition” by Abu-Laban, Scott, and Gowans that clearly holds policy implications. They found that among those who completed emergency medicine training as a third year of specialization through the College of Family Physicians Canada, a greater proportion had indicated on admissions to medical school many years earlier that emergency medicine was their first choice for a career in medicine compared to those who had not. They used data from over 1000 medical students accessed from a survey of 11 medical school classes from 8 Canadian universities anonymously linked to information from the Canadian Residency Matching Service. Policy makers should take notice.

In “Assessing the impact of a resident research program in general surgery,” Allen and her team of authors evaluated some of the outcomes of a formal program of resident research. Tracking resident research activities over a 10-year period, they found research productivity increased between time periods with a statistically significant increase in mean number of published abstracts. Many features of their program contributed to this successful implementation. Other programs could learn from their experiences.

Nagii and authors explored yet another deficiency in the non-medical expert education of medical students in their article, “Something’s missing from my education: Using a cross sectional survey to examine the needs and interests of medical students in Canada relating to their roles as teachers and educators.” Using an English language online questionnaire, they asked medical students from across Canada about their teaching experience, participation and/or awareness of teacher development at their school and awareness and/or interest in further training in medical education. They learned from the 169 respondents that while the majority expected or planned to teach when they entered practice, most reported no access to adequate training through medical school. I might also point out that training in education would also help medical students to teach each other more effectively and to be more informed critics of their own education.

In “Implications of not matching to a first-choice discipline: a family medicine perspective,” Woloschuk and his team examined differences between those who indicated family medicine was their first career choice and those who had prepared for and intended a career in another specialty. With an overall response rate of 47.2% (307/651), they found that the two groups were similar demographically, in preparedness for practice, and in lifestyle satisfaction and well-being. Those who chose family medicine first had significantly higher scores on perceptions of family medicine. Those findings deserve a thorough discussion of potential implications.

Veinot and her team of authors sought to understand how faculty and residents described their experiences of ambulatory care education within, between, and across disciplinary contexts. In “Faculty and resident perspectives on ambulatory care education: A collective case study of family medicine, psychiatry, and surgery,” they discovered some meaningful differences among specialties. In contrast to family medicine and psychiatry, surgery residents and faculty tended to equate ambulatory care with episodic experiences in clinic while surgery residents reported that ambulatory care education was less interesting and a much lower priority than operating. This should stir up some debate!

In “Time is of the essence: an observational time-motion study of internal medicine residents while they are on duty,” Leafloor and his team of authors concluded that a detailed recording of residents’ workflow was feasible and can therefore help describe the effects of changes to residency training. Interestingly they found that education activities accounted for 13% of on-duty time, while only 9% of the total on-duty time was spent in the presence of patients. Of the 17,714 events recorded, over 5% were interruptions. This made me wonder how productively I spend my time!

In “The development of national entrustable professional activities to inform the training and assessment of public health and preventative medicine residents,” Moloughney and his team of authors described the process they used to identify then achieve consensus on entrustable professional activities for a national Public Health and Preventive Medicine residency curriculum. They used a combination of workshops, a national online survey of public health and preventative medicine program directors, their national specialty committee, and competency-based education experts. Other programs and interest groups may find their work helpful.

Hughes and authors, in “Training the trainers: a survey of simulation fellowship graduates,” explored how the simulation fellowship experience of graduates throughout North America prepared them for their post-fellowship careers. Based on 35 responses, they concluded that the fellowship experiences adequately prepared the graduates for their post-fellowship simulation careers but that training in research and administration could be strengthened.

Bullock and his author team conducted a scoping review of 27 articles and reported their findings in “The prevalence and effect of burnout on graduate healthcare students.” They noted that graduate level healthcare students had higher levels of burnout than both age matched peers and the general population, and that burnout can affect their mental health, empathy, and professional conduct. The implications are not only personal (information to understand, recognize, and avoid burnout) but systemic (curricula and time for self-care).

In “The essential role of physician as advocate: how and why we pass it on,” Luft described the current state of advocacy in medical education and ways to foster the important skills of physician as advocate – another important non-medical expert role. Clearly there is a connection between the essential role of advocate and the valuable skill of argumentation!

Voll and Valiante, in “Watering CanMEDS flowers,” proposed that a major contributor to burnout is the increasing complexity and segregation of the medical profession and society as a whole. They claimed that the key to watering CanMEDS flowers and preventing physician burnout lies in getting back to the basics of human behaviour.

Fan and Aditya inspire us all with a vision of selfless service in their letter, “The pre-med ambition: is it worth it?” Youthful exuberance acknowledged, there are too many who enter medicine with an unrealistic and romantic ideal of the profession and end up being disappointed or eventually burnt out. Could some of what Fan and Aditya described actually contribute to this situation? Medical educators – especially those in admissions and recruiting – would do well to consider painting a more realistic panorama of medical careers.
